# Forming Limit Stress Diagram Prediction of Pure Titanium Sheet Based on GTN Model

**DOI:** 10.3390/ma12111783

**Published:** 2019-06-01

**Authors:** Tao Huang, Mei Zhan, Kun Wang, Fuxiao Chen, Junqing Guo, Yanyang Li, Zhuo Song, Luge Bai

**Affiliations:** 1 School of Materials Science and Engineering, Henan University of Science and Technology, Luoyang 471023, China; huangtao@haust.edu.cn (T.H.); wangkun@stu.haust.edu.cn (K.W.); guojq@haust.edu.cn (J.G.); 15737925685@163.com (Y.L.); 18848961105@163.com (Z.S.); 18739922732@163.com (L.B.); 2 Collaborative Innovation Center of Nonferrous Metals of Henan Province, Luoyang 471023, China; 3 State Key Laboratory of Solidification Processing, Northwestern Polytechnical University, Xi’an 710072, China; zhanmei@nwpu.edu.cn; 4 Henan Joint International Research Laboratory of Non-Ferrous Materials, Luoyang 471023, China

**Keywords:** GTN, finite element reverse calibration, hemispherical punch stretching test, FLD, FLSD

## Abstract

In this paper, the initial values of damage parameters in the Gurson–Tvergaard–Needleman (GTN) model are determined by a microscopic test combined with empirical formulas, and the final accurate values are determined by finite element reverse calibration. The original void volume fraction (*f_0_*), the volume fraction of potential nucleated voids (*f_N_*), the critical void volume fraction (*f_c_*), the void volume fraction at the final failure (*f_F_*) of material are assigned as 0.006, 0.001, 0.03, 0.06 according to the simulation results, respectively. The hemispherical punch stretching test of commercially pure titanium (TA1) sheet is simulated by a plastic constitutive formula derived from the GTN model. The stress and strain are obtained at the last loading step before crack. The forming limit diagram (FLD) and the forming limit stress diagram (FLSD) of the TA1 sheet under plastic forming conditions are plotted, which are in good agreement with the FLD obtained by the hemispherical punch stretching test and the FLSD obtained by the conversion between stress and strain during the sheet forming process. The results show that the GTN model determined by the finite element reverse calibration method can be used to predict the forming limit of the TA1 sheet metal.

## 1. Introduction

Titanium alloy sheets have gained extensive applications in the aviation and aerospace fields due to its superior mechanical and physical properties, such as high specific strength, superior thermal stability and good corrosion resistance [1,2,3,4,5]. The forming limit is an important performance index in the sheet metal forming process, which can reflect the maximum degree of deformation before the plastic instability of the sheet. It is the main basis for the development of the sheet metal forming process and die design [6]. For most metals, the experimental studies on forming limit have been carried out widely. The FLDs have been derived from an in-plane stretching test or a hemispherical punch-stretching test named Nakazima test, in which sheets are subject to biaxial stress. FLD has been proven to be an effective method in the analysis of sheet formability. However, the experimental and theoretical studies have also shown the maximum allowable limit strains rolling [7]. Kleemola and Pekkikangas [8] studied the forming limits of deep drawing quality steel, copper and brass, which followed uniaxial and equibiaxial pre-strain, and noticed the dependence of FLD on the magnitude and type of pre-strain. FLD has been considered to be accurate for proportional loading, where the ratio between the principal stresses remains constant in a forming process. In fact, the forming conditions are not equated to proportional straining sometimes. The ratio between the principal stress is observed to be nearly a constant during primary drawing processes in measurement and the finite element method mostly. The complex strain path is the main challenge in the study of forming limit [9]. Kleemola proposed FLSD to avoid the influence of strain path. FLSD is a failure criterion plotted with principal stress. It is always a unique curve under complex loading paths, which makes up for the limitation of strain paths in FLD and is more practical than FLD [10,11,12,13]. However, it is very difficult to measure the stress on the deformed sheet by experiment. During the forming test, the stresses are calculated by two methods, which are an incremental calculation according to the Levy-Mises flow law and finite element method. Thomas [14] and Xie et al. [15] showed that FLSD could be obtained by the transformation between strain and stress in the deformation of sheet metal. In addition, Uthaisangsuk et al. [16,17] used the finite element software ABAQUS to simulate and determine FLSDs. The FLSD of sheet metal is closely related to material damage in the finite element simulation analysis method. At present, the classical model of GTN is commonly used to describe material damage. However, it is rare to obtain the FLSD of sheet metal based on the GTN damage model. This inspires the author to use the GTN damage model to obtain FLSD of the sheet metal. Whether this method is feasible or not, it remains to be further studied.

The GTN model is the most widely used model for describing the plastic behavior of porous metal. It was originally proposed by Gurson and further improved by Tvergaard and Needleman. At present, the common methods for determining GTN damage parameters can be roughly summarized as the electron microscopy experimental analysis, representative volume element method and finite element reverse calibration method [18,19]. The volume fraction of voids can be obtained directly by an SEM experiment. However, due to the limited observation area, there are often some deviations between the simulation curve and the experimental curve. The representative volume element method obtains GTN parameters by finite element analysis of a single cell. However, there are some differences between the cell idealization and the actual material, and the analysis process needs to be given stress conditions, which is more complicated. The finite element reverse calibration method combines numerical simulation and experimental data to obtain a group or several groups of GTN damage parameters, which are consistent with the experimental data by constantly modifying the GTN parameters of materials.

In this paper, the hemispherical punch-stretching test is simulated by a plastic constitutive formula derived from the GTN model. The initial values of damage parameters in the GTN model are determined by SEM combined with empirical formulas, and the final accurate values are determined by finite element reverse calibration. The stress and strain are obtained at the last loading step before crack. The FLD and FLSD of the TA1 sheet under cold plastic forming conditions are plotted. Compared with the FLD obtained by the hemispherical punch stretching test and the FLSD obtained by the transformation between stress and strain, the results show a good agreement. It is verified that the parameters of the GTN model determined by finite element reverse calibration can be applied to the research of the forming limit for ductile metals and provide theoretical basis and technical guidance for production.

## 2. Materials and Methods

### 2.1. Materials

The annealed TA1 sheet with the thickness of 0.8 mm was provided by Shanxi Baoji Tengxin Titanium Industry Co., Ltd. The chemical composition of TA1 includes Ti, O, Fe, N, C and H. The corresponding mass fractions are 99.899%, 0.05%, 0.03%, 0.01%, 0.01% and 0.001%, respectively.

### 2.2. Methods

#### 2.2.1. Determining GTN Model Parameters with Finite Element REVERSE Calibration

The Gurson damage model is a single-stage void model, which considers the effect of void expansion on the plastic behavior of materials, but does not consider the interaction between the same-level voids and different-level voids [20]. Therefore, Tvergaard [21,22] and Needleman [23,24] considered the interaction between micropores based on the Gurson damage model, introduced the equivalent void volume fraction f*, and modified the Gurson damage model. The modified Gurson damage model is obtained, which is the GTN model:

(1) $$\phi ={\left(\frac{\sigma_{eq}}{\sigma_{y}}\right)}^{2}+2f^{*}q_{1}\cosh\left(\frac{3q_{2}\sigma_{H}}{2\sigma_{y}}\right)-\left[1+q_{3}{\left(f^{*}\right)}^{2}\right]=0$$

The f* can be expressed as:

(2) $$f^{*}=\left\{ \begin{array}{l} f\left(f\leq f_{c}\right)\\ f_{c}+\frac{f_{u}^{*}-f_{c}}{f_{u}^{*}+f_{c}}\left(f-f_{c}\right)\left(f_{c}<f^{*}<f_{F}\right)\\ f_{u}^{*}\left(f\geq f_{F}\right) \end{array}\right.$$

In the formula, *f_c_* is the critical voids volume fraction, *f_F_* is the voids volume fraction at the final failure, *q*_1_, *q*_2_ and *q*_3_ are the correction coefficients of yield surface equation, *f*_u_* = 1/*q*_1_.

As the volume fraction of the voids in the material increases, the plastic yield surface gradually decreases, that is, the material has a property of softening with material damage. When the material’s void volume fraction *f* = 0, the plastic yield surface is exactly the same as the classical plasticity theory’s Von Mises yield criterion. When the pore volume fraction *f* = 1, the plastic yield surface shrinks to a point where the material has broken. The nucleation and growth of the voids are the cause of the shrinkage of the plastic yield surface and the expansion of the plastic volume.

In the GTN model, the damage is considered to be isotropic, and the damage variable is represented by the void volume fraction. The void volume fraction change rate includes two parts:

(3) $$\overset{•}{f}={\overset{•}{f}}_{growth}+{\overset{•}{f}}_{nucleation}$$

In the formula, ${\overset{•}{f}}_{growth}$ is the volume fraction of voids growth and ${\overset{•}{f}}_{nucleation}$ is the volume fraction of voids nucleation.

Assuming that the matrix material is incompressible, the void growth is related to the macroscopic plastic volume deformation and described as:

(4) $${\overset{•}{f}}_{growth}=\left(1-f\right)\overset{•}{\varepsilon^{p}}$$

In the formula, $\overset{•}{\varepsilon^{p}}$ is equivalent to the plastic strain.

The volume fraction of voids nucleation is given by Chu and Needleman [25] based on the statistical method.

(5) $${\overset{•}{f}}_{nucleation}=\frac{f_{N}}{S_{N}\sqrt{2\pi }}\exp\left[-\frac{1}{2}{\left(\frac{\overset{•}{\varepsilon^{p}-\varepsilon_{N}}}{\varepsilon_{N}}\right)}^{2}\right]\overset{•}{\varepsilon^{p}}$$

In the formula, *f_N_* is the volume fraction of potential nucleated voids, *ε_N_* is the average equivalent plastic strain when voids nucleate, and *S_N_* is the standard deviation of the average strain when voids nucleate.

According to the above plastic constitutive equation, nine coefficients require to be identified in GTN model: *q*_1_, *q*_2_, *q*_3_, *ε_N_*, *S_N_*, *f*_0_, *f_c_*, *f_N_* and *f_F_* [26]. According to the results of Tvergaard [23], *q*_1_, *q*_2_, *q*_3_ can be fixed for most materials: *q*_1_ = 1.5, *q*_2_ = 1.0, *q*_3_ = 2.25. Since the initiation of voids begins at very low plastic strain levels, *ε_N_* and *S_N_* can take 0.2 and 0.1. For other damage parameters, the initial values are determined by SEM combined with the empirical formula, and the final accurate values are determined by finite element reverse calibration.

**Figure 1:**
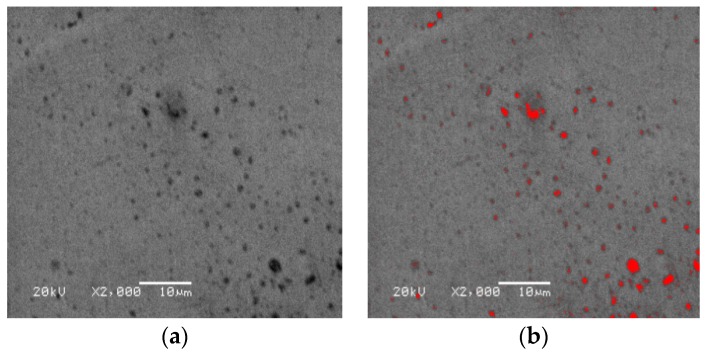
SEM photos of commercially pure titanium (TA1) (**a**) original morphology; (**b**) measurement results.

The initial voids in the material originate from a small number of original voids and second-phase particles in the material itself. The original morphology of TA1 used in this paper is shown in Figure 1a. In this paper, the Image-Pro Plus measurement software is used to measure *f*_0_. The measurement results are shown in Figure 1b. A correct selection of the measuring area (AIO) is one of the key steps to obtain the effective measurement data. Generally, the upper and lower area limits can be set in the segmentation tool to optimize the area of the selected voids. Meanwhile, multiple measurements can be considered, and the average values of several groups of data are selected to determine *f*_0_ of TA1. The measurement method is simple and convenient, but the upper and lower area limits need to be set manually in the measurement process, so there are still some errors in the measurement results, so it needs to be further optimized by the finite element reverse calibration method.

The image obtained by SEM is two-dimensional. The area obtained by the measurement software should be the area percentage of the voids, but it can be deduced from the mathematical formula that the area percentage of the voids is approximately equal to the volume percentage [27]. The analysis process is as follows: Assuming that the shape of the voids in the material is regular spherical. In any section of the material, the number of voids in the unit area is *N_S_*, and the number of voids in the unit length is *N_L_*, the radius of any void in the section is *r*. Then, the radius of the voids in any section of the material varies from 0 to *r*, and the cross-sectional area varies from 0 to *πr*^2^. The average area of the voids in the section can be expressed as follows:

(6) $$S=\frac{2\pi r^{2}}{3}$$

The radius *r* of the voids can be expressed as:

(7) $$r=\frac{2N_{L}}{\pi N_{S}}$$

The percentage of voids in the unit area is:

(8) $$f_{s}=\frac{2\pi r^{2}N_{S}}{3}$$

The volume percentage of voids in the unit volume is:

(9) $$f_{v}=\frac{8N_{L}^{2}}{3\pi N_{S}}$$

Bringing the formula (2) into the formula (3):

(10) $$f_{s}=\frac{2\pi r^{2}N_{S}}{3}=\frac{2\pi }{3{\left(2N_{L}/\pi N_{S}\right)}^{2}N_{S}}=f_{v}$$

From the above deduction, it can be seen that the area percentage of the material voids is approximately equal to the volume percentage of voids. Therefore, the volume percentage of voids can be determined by calculating the area percentage of voids with image analysis and measurement software.

*f_c_* can be determined by the following empirical formula suggested by Benseddiq [28]:

(11) $$f_{c}=0.0186Ln\left(f_{0}\right)+0.1508$$

*f_F_* can be determined by the following empirical formula suggested by Zhang [29]:

(12) $$f_{F}=0.15+2f_{0}$$

The initial values of damage parameters determined by SEM and empirical formulas have a certain degree volatility. In order to obtain more accurate damage parameters, the damage parameters of the GTN model are accurately determined by the combination of notched uniaxial tensile test and finite element simulation in this paper. Firstly, the load-displacement (*F*-*s*) curve is extracted from the numerical simulation results of the notched uniaxial tensile test. The *F*-*s* curve of the notched uniaxial tensile test is compared with the *F*-*s* curve obtained by numerical simulation. In addition, the damage parameters are continuously adjusted so that the difference between the *F*-*s* curve obtained by numerical simulation and that measured by the notched uniaxial tensile test is the smallest. That is, the objective function *Q* is the minimum value, so that the final accurate damage parameters of the GTN model of TA1 can be obtained.

(13) $$Q=\frac{1}{n}\sum_{i=1}^{n}\left|\frac{F_{i}^{\exp}-F_{i}^{sim}}{F_{i}^{\exp}}\right|$$

In the formula, *n* is the number of sampling points on the *F*-*s* curve, *F_i_^exp^* and *F_i_^sim^* are the load values of experimental and finite element simulation at sampling point i, respectively.

The finite element reverse calibration method has the defects of uncertainty and large computational quantity. Therefore, it is necessary to select the appropriate optimization algorithm to accelerate the reverse efficiency and avoid the local optimal solution. The objective function used in this paper is more complex, which is the average of the error functions of n sampling points, and the finite element reverse calibration results hope that the error of each sampling point is relatively small. The NSGA-II [30] optimization algorithm has low computational complexity and has an elite strategy. The exploration performance is good and the accuracy of each sampling point can be guaranteed. Therefore, the NSGA-II optimization algorithm is adopted in this paper. The nine groups GTN model damage parameters of TA1 in the finite element reverse calibration process are shown in Table 1.

**Figure 2:**
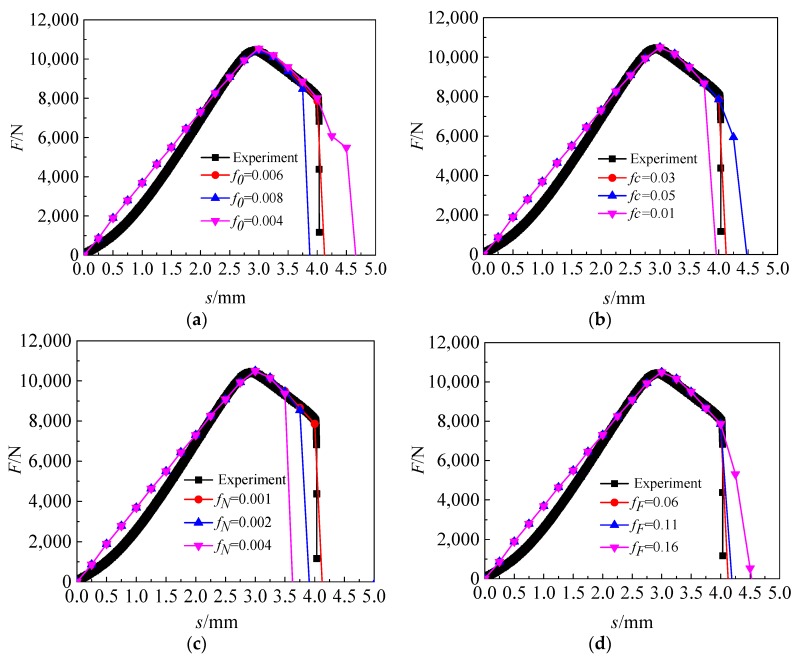
Influence of damage parameters on load-displacement (*F*-*s*) curves (**a**) change void volume fraction (*f*_0_); (**b**) change critical void volume fraction (*f_c_*); (**c**) change volume fraction of potential nucleated voids (*f_N_*); (**d**) change void volume fraction at the final failure (*f_F_*).

As shown in Figure 2a, it can be found that when the value of *f*_0_ increases, the end of the *F*-*s* curves move to the left. When the value of *f*_0_ decreases, the end of the *F*-*s* curves move to the right. This is because the value of *f*_0_ represents the original voids volume fraction of the material. The large value of *f*_0_ indicates the more original micro-voids in the material, and then the overall bearing capacity of the material decreases. On the contrary, the small value of *f*_0_ indicates the less original micro-voids in the material, and then the overall bearing capacity of the material is improved.

As shown in Figure 2b, it can be found that when the value of *f_c_* increases, the end of the *F*-*s* curves move to the right. When the value of *f_c_* decreases, the end of the *F*-*s* curves move to the left. This is because the value of *f_c_* represents the critical voids volume fraction of the material. The larger the value of *f_c_* is the later time at which the material voids begin to polymerize is, and then the overall bearing capacity of the material is improved. On the contrary, the smaller the value of *f_c_* is the earlier time at which the material voids begin to polymerize is, and then the overall bearing capacity of the material decreases.

As shown in Figure 2c, it can be found that when the value of *f_N_* increases, the end of the *F*-*s* curves move to the left. When the value of *f_N_* decreases, the end of the *F*-*s* curves move to the right. This is because the value of *f_N_* represents the volume fraction of potential nucleated voids of the material, which directly affects the incremental process of voids evolution. The larger the value of *f_N_*, the greater the volume fraction of new voids produced by material nucleation, the lower overall bearing capacity of material. On the contrary, the smaller the value of *f_N_*, the smaller the volume fraction of new voids produced by material nucleation, the higher overall bearing capacity of materials.

As shown in Figure 2d, it can be found that when the value of *f_F_* increased, the time at which the material finally breaks is delayed, when the value of *f_F_* is decreased, the time at which the material finally breaks is advanced. The larger the difference between the value of *f_F_* and *f_c_*, the smaller the load drop rate when the volume fraction of the voids exceeds *f_c_*. On the contrary, the smaller the difference between the value of *f_F_* and *f_c_*, the greater the load drop rate when the volume fraction of the voids exceeds *f_c_*.

**Figure 3:**
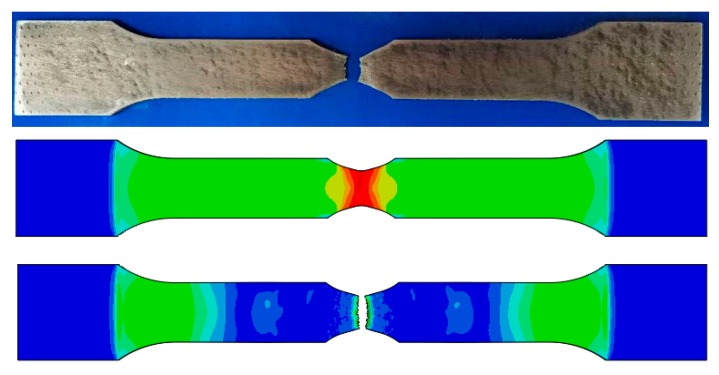
Comparison of notch uniaxial tensile test results and simulated results.

**Figure 4:**
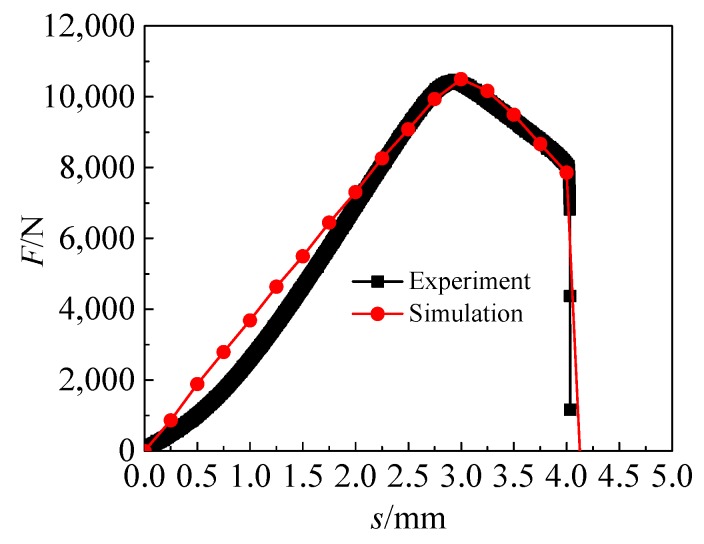
*F*-*s* curves obtained from experiment and simulation.

The final damage parameters of the GTN damage model of TA1 are obtained by the above method as shown in the first group in Table 1. Figure 3 is the comparison of the notch uniaxial tensile test and simulation of TA1. Figure 4 shows the TA1 *F*-*s* curve obtained by finite simulation based on the final damage parameters of the GTN damage model, which is in good agreement with the uniaxial tensile test results.

#### 2.2.2. Establishment of Finite Element Model

The finite element model of hemispherical punch stretching test is established based on the ABAQUS finite element simulation software. The strain and stress near the crack were calculated by simulation, and the FLD and FLSD of TA1 are constructed.

**Figure 5:**
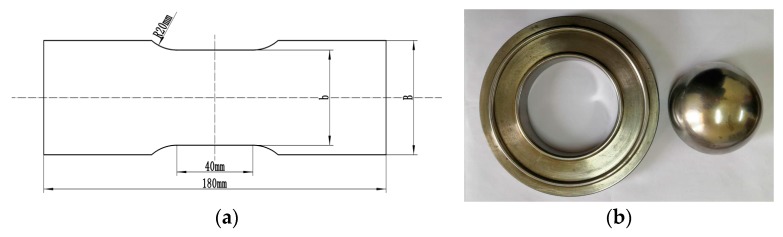
The dimensions of specimens and moulds (**a**) stepped specimen; (**b**) punch and die.

**Figure 6:**
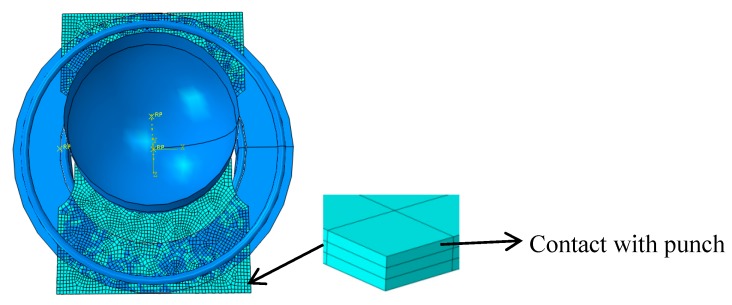
Finite element model.

(1) Geometry size: The experimental for obtaining FLD reference standard GB/T 15825.8-2008 [31], The length of stepped specimens are 180 mm, and the widths (B) are 120 mm, 100 mm, 80 mm, 60 mm, 40 mm and 20 mm, respectively, as shown in Figure 5a. The dimensions of square specimens are 180 mm × 180 mm. The diameter of the hemispherical punch is 100 mm and the aperture of the die is 110 mm, as shown in Figure 5b.
(2) Material parameters: Density is 4.51 g/mm^3^, elastic modulus is 106.605 GPa, and Poisson’s ratio is 0.34.
(3) Contact friction: The contact between stepped specimens and moulds is modeled by surface-to-surface contact. The Coulomb friction coefficient is set to *µ* = 0.25. The Coulomb friction coefficients between square specimens and moulds are 0.1, 0.15, 0.2 and 0.25, respectively.
(4) Boundary conditions: The blanking force is 140 KN when the sample width is 20 mm and 40 mm, the blanking force is 160 KN when the sample width is 60 mm and 80 mm, and the blanking force is 180 KN when the sample width is 100 mm and 120 mm. The blanking force is 220 KN when the sample is a square specimen, and the bulging speed is 0.2 mm/s.
(5) Mesh: Since material failure always develops on the free material surface, the sheet metal geometry is also meshed in three element layers (contact with punch, middle layer and free surface). According to the above key techniques, the finite element model is shown in Figure 6.

The stress of the layer of contact with punch is usually unstable. However, the stress in the element at the same location but at the free surface generally shows a much more stable process [32]. Therefore, the analyses of the strain and the stress are selected in the free surface layer element of the bulging specimen.

## 3. Results

**Figure 7:**
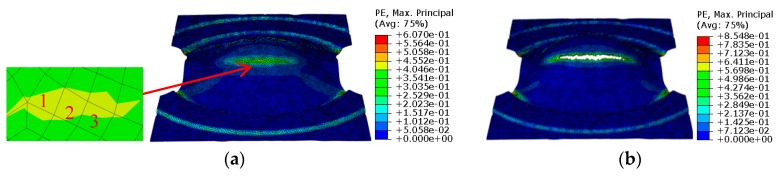
Strain states before and after fracture (**a**) before fracture; (**b**) after fracture.

**Figure 8:**
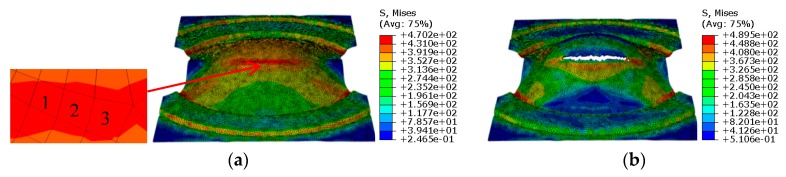
Stress states before and after fracture (**a**) before fracture; (**b**) after fracture.

**Figure 9:**
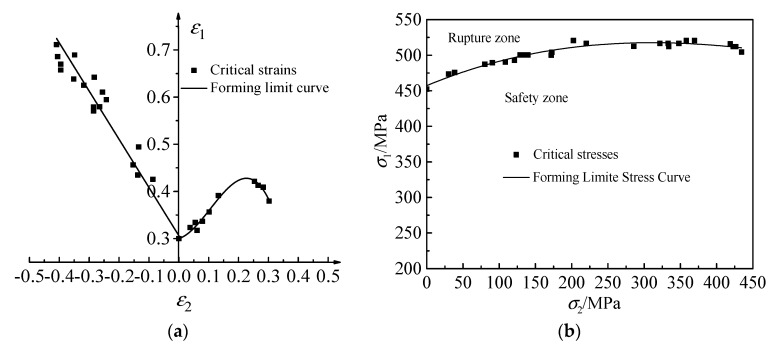
Forming limit diagram (FLD) and forming limit stress diagram (FLSD) of TA1 sheet (**a**) FLD; (**b**) FLSD.

Based on the identified parameters of the GTN model, the simulation of hemispherical punch stretching tests was carried out for the stepped specimens with a width of 120 mm, 100 mm, 80 mm, 60 mm, 40 mm and 20 mm, and the square specimens with a length and width of 180 mm × 80 mm. Due to the fact that the simulation process and the treatment method of the simulation results are the same, the simulation results of the stepped specimens with a width of 120 mm are taken as an example for subsequent analysis. The strain states and stress states before and after the fracture in the sheet of the width of 120 mm are shown in Figure 7; Figure 8. Three critical elements located in the necking zone at the free surface layer in one sample are selected at the last loading step without cracks, the strain and stress difference between every two elements is within 10%. The average values of principal strains among the three elements, shown in Figure 7a, are calculated and regarded as the critical principal strain for one sample. FLD is plotted according to the critical maximal principal strain *ε*_1_ and the critical middle principal strain *ε*_2_ before crack on all widths, shown in Figure 9a. The average values of principal stresses among the three elements, shown in Figure 8a, are calculated and regarded as the critical principal stresses for one sample. FLSD is plotted according to the critical maximal principal stress *σ*_1_ and the critical middle principal stress *σ*_2_ before crack on all widths, shown in Figure 9b.

**Figure 10:**
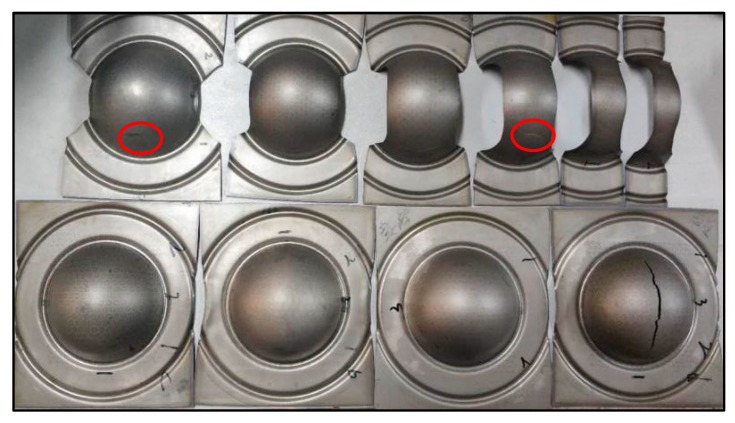
Hemispherical punch stretching tests for part of specimens.

The experimental conditions in the verification experiment are identical to those in Section 2.2.2. The experimental results are shown in Figure 10.

## 4. Discussion

**Figure 11:**
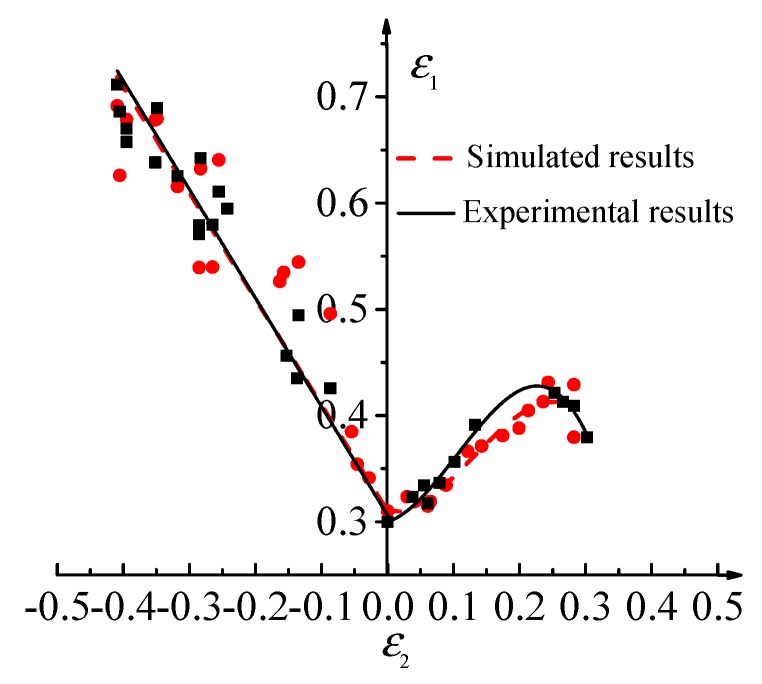
Comparison of FLD of TA1 between simulated and experimental results.

**Figure 12:**
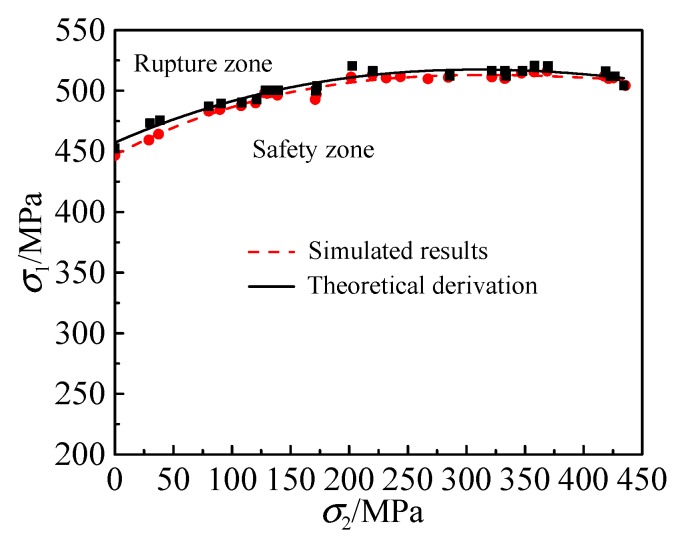
Comparison of FLSD of TA1 between simulated and theoretical derivation.

The plane strain coordinate system is established by taking the ε_2_ as abscissa and the ε_1_ as ordinate. The ε_1_ and ε_2_ measured by each sample are labeled in the strain coordinate system. FLD of the TA1 sheet can be obtained by optimization based on the least square method. The critical strains can be transformed into the critical stresses based on the isotropic strengthening criterion and the stress-strain relationship in the incremental theory, thereby the FLSD of the TA1 sheet under the plastic deformation condition can be established [33]. The FLDs of the TA1 sheet obtained by the experiment and finite element simulation are shown in Figure 11. The maximum relative error of the two curves is 8.98%. The FLSDs of the TA1 sheet obtained by theoretical derivation and finite element simulation are shown in Figure 12. The maximum relative error of the two curves is 7.21%. The main reasons for the errors are as follows: (1) The setting of Coulomb friction coefficient in the simulation process may not fully meet the actual situation, which will lead to the deviation of the simulation results from the experimental results. (2) The measurement of the strain in the experiment process is based on the grid rubbing technology. The measurement error of the system is about 2%, which will lead to the deviation of the simulation results from the experimental results. (3) The minimum value of the objective function *Q* is 0.048 in the finite element reverse calibration process. The existence of this error will also cause some difference between the experimental results and the simulation results. It is considered that the error is within a reasonable range and the simulation results are reliable based on the above reasons.

## 5. Conclusions

(1) The parameters in the GTN damage model of TA1 are determined by finite element reverse calibration. *f*_0_, *f_N_*, *f_c_* and *f_F_* are assigned as 0.006, 0.001, 0.03 and 0.06, respectively. The GTN model parameters obtained from finite element reverse calibration are proved available for ductile metals by comparing the force-displacement curves obtained by the finite element simulation and notched uniaxial tensile test.
(2) The GTN damage model parameters obtained by finite element reverse calibration are used as the failure criterion combined with the finite element analysis method to obtain the FLD and FLSD of the TA1, which is in a good agreement with the FLD and FLSD of the TA1 obtained by experimental and theoretical derivation. It is verified that the GTN model parameters determined by the finite element reverse calibration can be used to study the forming limit of ductile metals, and provide theoretical basis and technical guidance for actual production.

## Figures and Tables

**Table 1:** Nine groups damage parameters of TA1 in the finite element reverse calibration process.

Group	*f*_0_	*f_c_*	*f_N_*	*f_F_*	*ε_N_*	*S_N_*	*q*_1_	*q*_2_	*q*_3_
1	0.006	0.03	0.001	0.06	0.2	0.1	1.5	1.0	2.25
2	0.006	0.03	0.004	0.06					
3	0.006	0.03	0.002	0.06					
4	0.006	0.05	0.001	0.06					
5	0.006	0.01	0.001	0.06					
6	0.006	0.03	0.001	0.11					
7	0.006	0.03	0.001	0.16					
8	0.004	0.03	0.001	0.06					
9	0.008	0.03	0.001	0.06					
